# Effects of Rapid Thermal Annealing on the Structural, Electrical, and Optical Properties of Zr-Doped ZnO Thin Films Grown by Atomic Layer Deposition

**DOI:** 10.3390/ma9080695

**Published:** 2016-08-13

**Authors:** Jingjin Wu, Yinchao Zhao, Ce Zhou Zhao, Li Yang, Qifeng Lu, Qian Zhang, Jeremy Smith, Yongming Zhao

**Affiliations:** 1 Department of Electrical Engineering and Electronics, University of Liverpool, Liverpool L69 3GJ, UK; Jingjin.wu@liverpool.ac.uk (J.W.); Yinchao.zhao@liverpool.ac.uk (Y.Z.); qifeng@liverpool.ac.uk (Q.L.); J.S.Smith@liverpool.ac.uk (J.S.); 2 Department of Electrical and Electronic Engineering, Xi’an Jiaotong-Liverpool University, Suzhou 215123, China; 3 Department of Chemistry, Xi’an Jiaotong-Liverpool University, Suzhou 215123, China; Li.Yang@xjtlu.edu.cn (L.Y.); Qian.Zhang@xjtlu.edu.cn (Q.Z.); 4 Key Lab of Nanodevices and Applications, Suzhou Institute of Nano-Tech and Nano-Bionics, Chinese Academy of Sciences (CAS), Suzhou 215123, China; ymzhao2009@sinano.ac.cn; 5 University of Chinese Academy of Sciences, Beijing 100049, China

**Keywords:** Zr-doped ZnO, atomic layer deposition, rapid thermal annealing, red-shift

## Abstract

The 4 at. % zirconium-doped zinc oxide (ZnO:Zr) films grown by atomic layer deposition (ALD) were annealed at various temperatures ranging from 350 to 950 °C. The structural, electrical, and optical properties of rapid thermal annealing (RTA) treated ZnO:Zr films have been evaluated to find out the stability limit. It was found that the grain size increased at 350 °C and decreased between 350 and 850 °C, while creeping up again at 850 °C. UV–vis characterization shows that the optical band gap shifts towards larger wavelengths. The Hall measurement shows that the resistivity almost keeps constant at low annealing temperatures, and increases rapidly after treatment at 750 °C due to the effect of both the carrier concentration and the Hall mobility. The best annealing temperature is found in the range of 350–550 °C. The ZnO:Zr film-coated glass substrates show good optical and electrical performance up to 550 °C during superstrate thin film solar cell deposition.

## 1. Introduction

ZnO is believed to be a promising indium tin oxide (ITO) substitute material due to its abundance and large optical band gap, and because it can easily be doped to degeneracy. The high carrier density in the impurity-doped ZnO film will slightly increase the band gap due to the Burstein–Moss effect [1]. N-type dopants that are commonly used in ZnO are Al [2,3,4] and Ga [5,6,7,8], while trials on B [9,10], In [11,12], Ge [13], Ti [14], Zr [15,16,17,18,19,20,21,22,23,24,25,26], and Hf [27] are also reported. Zirconium ion dopant attracts worldwide attention due to its physical nature. The ionic size of Zr^4+^ is comparable to that of Zn^2+^ which helps to minimize the lattice distortion [25]. Moreover, zirconium-doped zinc oxide (ZnO:Zr) films are stable at high temperature (<800 K) compare to ITO (<420 K) [28]. Stability at a high temperature is crucial for electronic device fabrication, especially for the fabrication of thin film solar cells in superstrate configuration.

Various technologies have been used to dope Zr on ZnO, including magnetron sputtering [15,16,17], spray pyrolysis [19,20], sol-gel [23], pulsed laser deposition [21,22], and atomic layer deposition (ALD) [24,25]. ALD was introduced in ZnO:Zr thin films’ deposition due to its ability to accurate control the thin film composition and thickness. In principle, ALD proceeds in layer-by-layer growth, hence the doping concentration can be controlled by the proportion of dopant and bulk layer. 

Recently, comprehensive reports about ALD-derived ZnO:Zr thin film were presented. Lin et al. [24] first demonstrated ZnO:Zr films by ALD. By varying the doping concentration, they obtained films with low resistivity (1.3 × 10^−3^ ohm·cm), high carrier concentration (2.2 × 10^20^ cm^−3^), and high transparency (92%) in the visible spectrum [24]. Following on their research, Herodotou et al. [25] have further investigated the effects of doping concentration and thin film thickness on ZnO:Zr films and obtained similar results. They illustrated that ZnO:Zr film with 4.8 at. % doping results in reduced resistivity (1.44 × 10^−3^ ohm·cm), increased carrier density (3.81 × 10^20^ cm^−3^), and increased optical band gap to 3.5 eV [25]. Both of them examined the effect of doping concentration, however, the investigation of the rapid thermal annealing (RTA) process on ALD-derived ZnO:Zr film is lacking. It is known that the stability of transparent conducting oxide (TCO) films is of great importance since TCO-coated glass will be used as the substrate in superstrate thin film solar cells and will suffer high temperature during solar cell fabrication. ZnO:Zr film is predicted as the most likely substrate candidate in thin film solar cell fabrication. Therefore, robust and good performance of ZnO:Zr film withstanding high temperature is of major strategic importance for solar cells fabrication. In this work, ZnO:Zr films with 4 at. % doping were deposited by ALD, then the samples were subjected to RTA for 3 min. The structural, optical, and electrical characterizations were performed to obtain a better understanding of the stability of ZnO:Zr film under various temperatures.

## 2. Results and Discussion

### 2.1. Structural Properties

**Figure 1:**
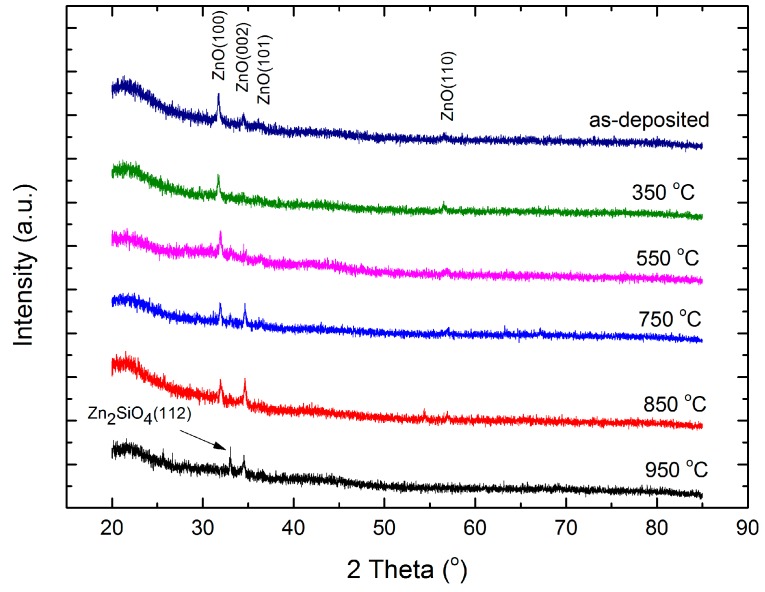
X-ray diffraction (XRD) pattern of ZnO:Zr thin films annealed at different temperatures.

The structural properties of the annealed ZnO:Zr films were investigated by X-ray diffraction (XRD). Figure 1 shows the XRD patterns of the as-deposited film and films annealed at various temperatures. As shown in Figure 1, the as-grown ZnO:Zr film is of polycrystalline structure and belongs to the hexagonal wurtzite structure. No other oxides were detected, suggesting that Zr^4+^ either replaced Zn^2+^ site, occupied at the interstitial site in the hexagonal lattice structure, or that amorphous ZrO_2_ phases were formed at the non-crystalline region in the grain boundary. It also indicated that the replacement and occupation of Zr^4+^ does not change the hexagonal wurtzite structure of the ZnO film. Additionally, unlike doped ZnO films deposited by other techniques, the ZnO:Zr films grown by ALD exhibit three diffraction peaks which correspond to the ZnO (100), (002), and (101) peaks. The prominent (100) diffraction peak in our work agreed with the result reported by Pung et al. [29]. They found that the intensity of the (100) peak is dominant for undoped ZnO films when depositing in the temperature range of 155–200 °C by ALD. This can be explained by the fact that while ZnO films grown using other techniques have the fastest growth rate along the c-axis [20,21], ZnO:Zr films grown by ALD exhibit a small tensile component parallel to the c-axis instead. The lattice constants were calculated to further understand the effect of the deposition technique on the thin film structural properties. The lattice constants a and c can be obtained for the symmetric (100) and (002) planes by the formula [28]:

(1) $$\frac{1}{d^{2}}=\frac{4}{3}\left(\frac{h^{2}+hk+k^{2}}{a^{2}}\right)+\frac{l^{2}}{c^{2}}$$

where *d* is the interplanar spacing and *h*, *k*, and *l* are crystal orientations.

**Figure 2:**
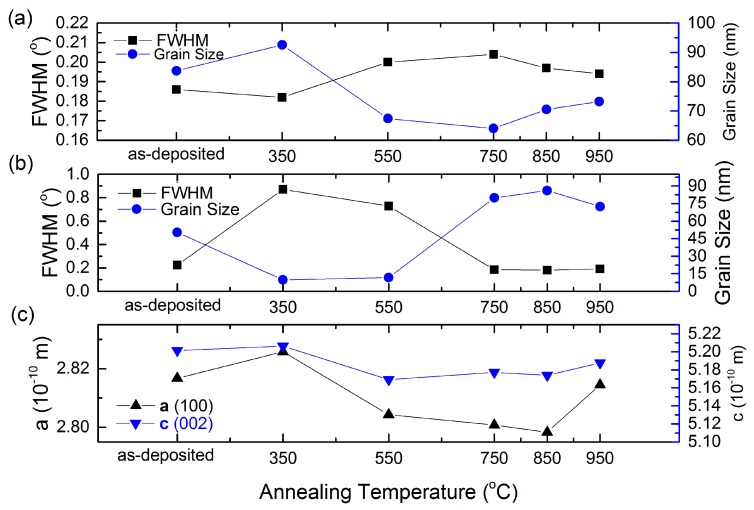
Variation of grain size, full-width at half-maximum (FWHM) and lattice constant with respect to annealing temperature: (**a**) FWHM and grain size with respect to annealing temperature for peak (100) in the XRD pattern; (**b**) FWHM and grain size with respect to annealing temperature for peak (002) in the XRD pattern; (**c**) lattice constant with respect to annealing temperature.

By calculating the c-axis lattice parameter from the XRD (002) plane, it was found that the lattice parameter for the ZnO films in our work is 5.19 Å, which is smaller than relaxed bulk ZnO (c = 5.207 Å) and sputtered ZnO films (c = 5.216 Å), as shown in Figure 2 [27]. Since the radius of Zr^4+^ (0.59 Å) is similar to that of Zn^2+^ (0.6 Å), the variation of lattice constants cannot be explained by the difference between the radii. This may be due to the low tensile strain paralleled to the c-axis during thin film deposition.

All three primary diffraction peaks, namely (100), (002), and (101) were still dominant when increasing the temperature up to 750 °C, which implies that all the films appear to crystallize equiaxially. When the temperature was further increase to 850 °C, the (101) peak disappeared. At 950 °C, the (100) and (002) peaks were no longer observed and the Zn_2_SiO_4_ characteristic peak at 33.027° appeared in the diffraction pattern.

The grain size (D) of the corresponding films can be calculated from the full-width at half-maximum (FWHM) of the (100) peak by Scherrer’s equation [27]:

(2) $$D=\frac{k\lambda }{\beta cos\theta }$$

where λ is the X-ray wavelength of the Cu Kα, θ is the Bragg diffraction angle, and β is the FWHM of the (100) peak in radians. As seen in Figure 2a, the grain size increased and reached a peak value at RTA 350 °C, then decreased to a minimum of 70 nm at 750 °C. At higher temperatures of 850 °C and 950 °C, we see the grain size increasing again. The initial increase might be due to more activation energy provided to the atoms, leading to larger grains. However, at higher temperatures, the zirconium interstitials may diffuse and segregate to the non-crystalline region in the grain boundary, which will cause crystal disorder and thus shrink the grain size. It is known that the ALD-derived ZnO:Zr thin films are composed of multilayers of ZnO and one zirconium oxide layer in one supercycle. Since ZrO_2_ and ZnO have such different structures and that monoclinic/cubic/tetragonal are similarly different, the formation of ZrO_2_ layers will be suppressed in the hexagonal wurtzite structure ZnO. Hence, most Zr^4+^ ions act as Zn^2+^ substitution or interstitials. With high annealing temperature, more activation energy is provided to the Zr^4+^ atoms, which results in a diffusion of the ions into the ZnO matrix and the ZnO grain boundary. When the annealing temperature further increases to 850 °C, the grain size increases again. The main reason for the grain size growth is probably due to ZnO grain growth and the formation of Zn_2_SiO_4_.

**Figure 3:**
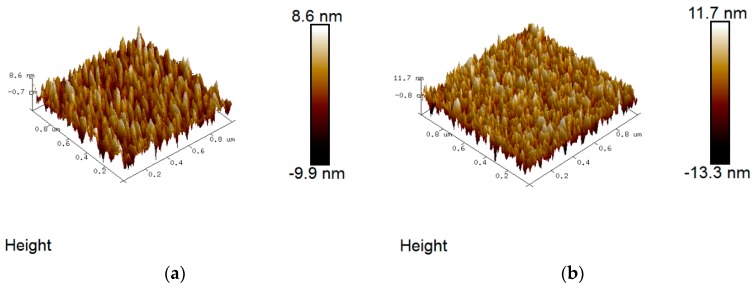

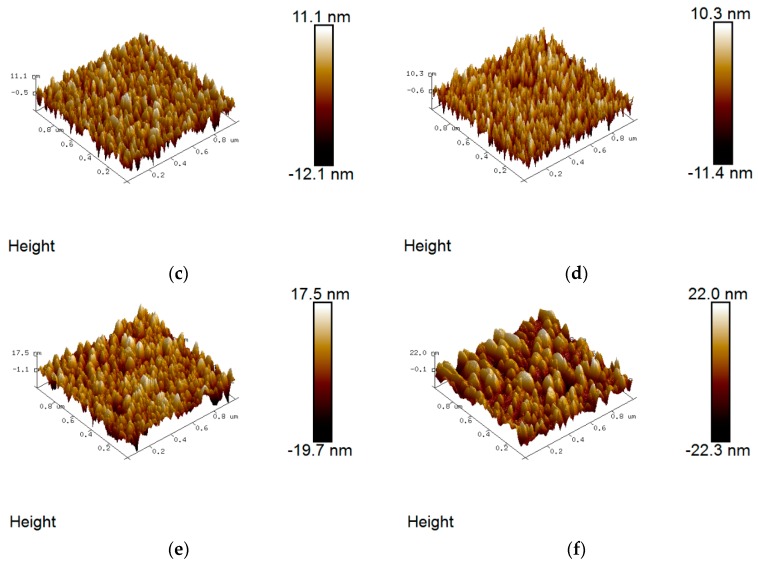
Atomic force microscopy (AFM) images of ZnO:Zr films grown on Si (100): (**a**) as-deposited; (**b**) 350 °C; (**c**) 550 °C; (**d**) 750 °C; (**e**) 850 °C; and (**f**) 950 °C.

**Figure 4:**
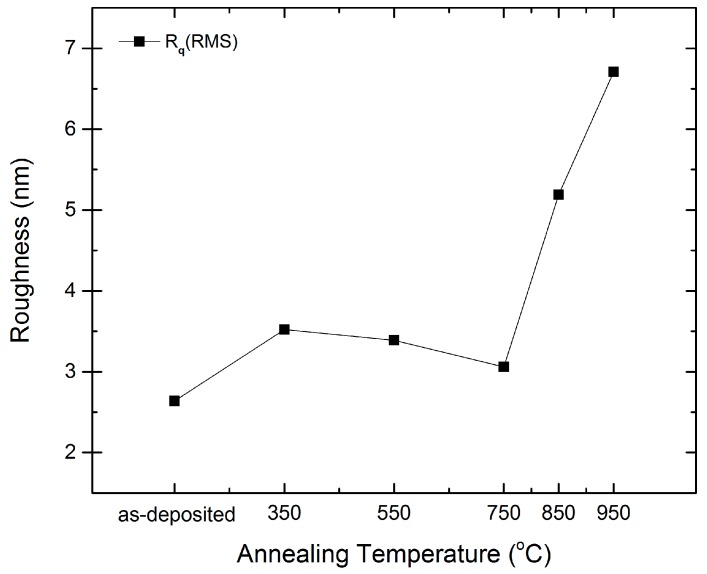
Surface roughness of the as-deposited and annealed ZnO:Zr thin films grown on Si (100).

Since transparent conducting oxide (TCO) coated glass is used as substrate in thin film solar cell fabrication, the smoothness of the surface affects the thin films’ deposition at the subsequent stage and thus device performance. Hence, it is important to study the surface morphology as a function of the annealing temperature of the ZnO:Zr films. Figure 3 shows the atomic force microscopy (AFM) images of the as-deposited and annealed ZnO:Zr films on a scanning area of 1 μm × 1 μm. No pin holes were observed in the micrographs, indicating successful deposition of compact films. As seen in Figure 4, the root mean square roughness (RMS) of the as-deposited films is about 2.5 nm, which is in good agreement with that reported by Yan et al. [30]. The grain size of the annealed ZnO:Zr films slightly decreases, as expected, before significantly increasing again at 850 °C. It can be observed in Figure 3 that the grain size parallel to the surface is getting smaller, while the grain size vertical with the substrate is larger. The former reflects the (100) crystalline orientation, and the latter corresponds to the (002) crystalline orientation. Hence, the transformation of the prominent crystalline orientation from (100) to (002) is attributed to RTA process, which is consistent with the hypothesis obtained from the XRD patterns.

### 2.2. Optical Properties

**Figure 5:**
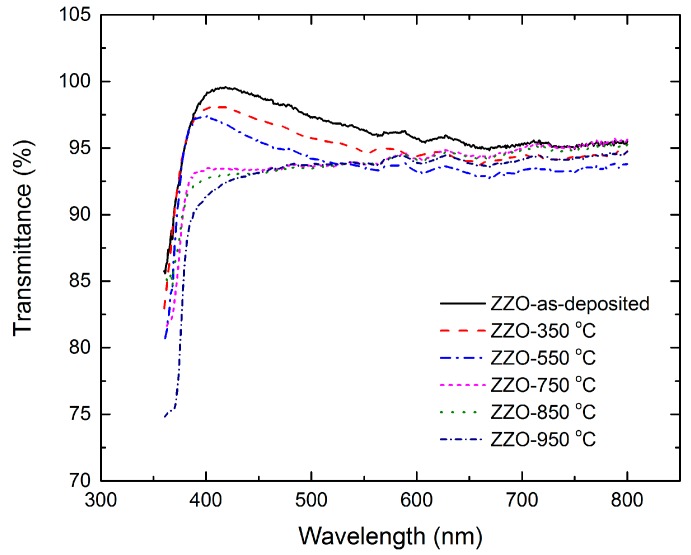
Optical transmittance as a function of wavelength for as-deposited and annealed ZnO:Zr films.

**Figure 6:**
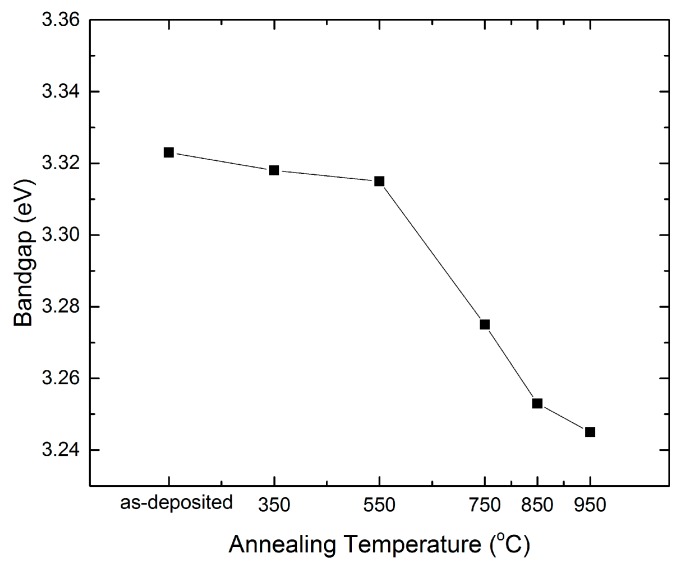
Optical band gap (Eg) of ZnO:Zr thin films at various annealing temperatures.

Since the ZnO:Zr film-coated glass is expected to be used as substrate for thin film solar cell fabrication, it is necessary to investigate the optical properties of ZnO:Zr films under various RTA treatments. Figure 5 shows the optical transmittance in the wavelength region 350–800 nm of as-deposited and annealed ZnO:Zr films. It is clear that the films show an average transmittance of over 92% in the visible wavelength. Also, it is important to note that with the increase of the annealing temperature, the edge of the transmission spectrum was red-shifted. The optical band gap ($E_{g}$) was determined by extrapolation of the plot of α^2^ versus hv [31].

(3) $$a\left(hv\right)=A{\left(hv-E_{g}\right)}^{n}$$

where α is the absorption coefficient, A is a constant, hv is the photon energy, $E_{g}$ is the optical band gap, and *n* = 1/2 for direct band gap material. Figure 6 shows the optical band gap of the ZnO:Zr films under various RTA treatments. The band gap of the as-deposited ZnO:Zr film is 3.31 eV, in agreement with the 3.30 eV reported by Lin et al. [24]. The band gap keeps almost constant up to 550 °C, then, it decreases to 3.245 eV after the 950 °C treatment. The band gap narrowing effect (EBGN) is believed to be a result of many body effects, and is expressed as [25]:

(4) EBGN=e22πε0εr(3πn)13

where $\varepsilon_{0}$ is permittivity of vacuum, $\varepsilon_{r}$ is relative permittivity, and n is the carrier concentration. The optical band gap is proportional to (n)13. In Figure 6, the optical band gap decreases with the increase of the RTA temperature. Therefore, it is expected that carrier concentration will decrease when the annealing temperature is increasing.

### 2.3. Electrical Properties

**Figure 7:**
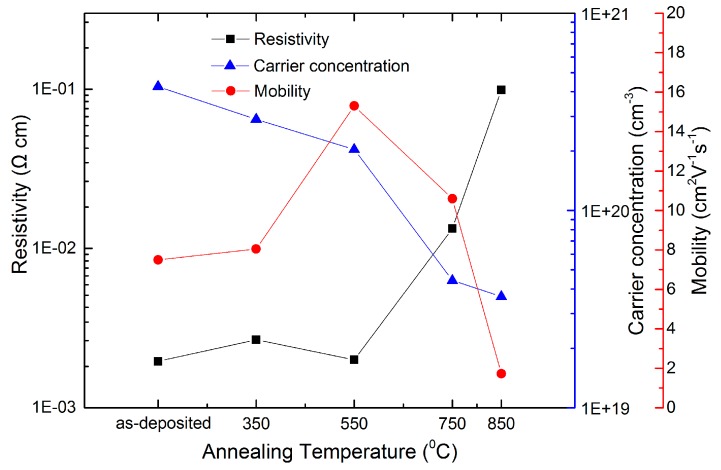
The resistivity, carrier concentration, and mobility of the ZnO:Zr films as a function of annealing temperature.

The electrical properties of the ZnO:Zr films were characterized by Hall measurements using the van der Pauw configuration at room temperature. The change of electrical properties with the annealing temperature is illustrated in Figure 7. The resistivity keeps almost constant at annealing temperatures up to 550 °C, then increases sharply from 1.27 × 10^−3^ to 0.97 × 10^−1^ ohm·cm due to the rapid decrease of the carrier concentration and Hall mobility. Finally, it becomes an insulator after the 950 °C treatment. For the ZnO:Zr films, carriers come from oxygen vacancies, Zn ions on interstitial lattice sites, and the substitution of Zr^4+^ on the Zn^2+^ site. It is reported that there are far fewer oxygen vacancies in the doped films than those in the undoped film [27]. Hence, the carrier concentration is mainly attributed to the Zr^4+^ doping. As previously mentioned, a diffusion of Zr^4+^ interstitials to the non-crystalline region in the grain boundary results in a decrease of carrier concentration. This phenomenon is also predicted from the band gap narrowing equation. An alternative explanation could be zinc evaporation during the annealing process leading to the decrease in carrier concentration. The mobility of the as-deposited films is 7.8 cm^2^·V^−1^·S^−1^, and it rises to 15.7 cm^2^·V^−1^·S^−1^ after the 550 °C RTA treatment. Then the mobility decreases dramatically to 1.6 cm^2^·V^−1^·S^−1^ at 850 °C. As known, grain boundary scattering and ionized impurity scattering affect the mobility of the film. The resultant mobility follows the equation [17]:

(5) $$\frac{1}{\mu }=\frac{1}{\mu_{GB}}+\frac{1}{\mu_{IS}}$$

where µ is the resultant mobility, $\mu_{GB}$ is the grain boundary scattering affected mobility, and $\mu_{IS}$ is the ionized impurity scattering affected mobility. The initial increase of the Hall mobility indicates that the grain growth is dominant over other factors. When the annealing temperature increases above 550 °C, the Hall mobility decreases due to the increase of grain boundary scattering.

## 3. Materials and Methods 

Thin ZnO:Zr films were grown by an MNT F-200 system on quartz glass and virgin test grade Si (100) substrate, respectively. The optical properties and electrical properties of ZnO:Zr films were investigated on the quartz glass sample, while ZnO:Zr films on silicon substrate were used to characterize the thin film morphology.

**Figure 8:**
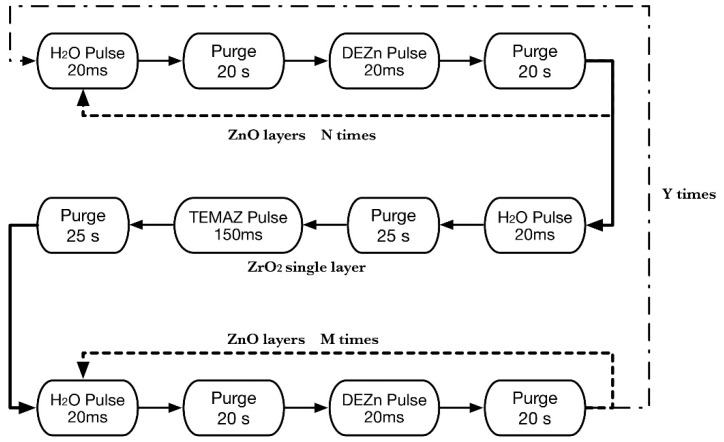
Schematic of the atomic layer deposition (ALD) deposition sequence used to deposit ZnO:Zr thin films.

The substrates were firstly ultrasonically cleaned in isopropanol, acetone, alcohol, and deionized (DI) water for 10 min each. Then, each substrate was dried using high purity nitrogen. Diethyl-zinc (DEZn) and tetrakis-ethylmethylamino zirconium (TEMAZ) were used as the Zn and Zr sources, respectively, and DI water acted as an oxidizing agent. These precursors and an oxygen source were alternately introduced into the growth chamber with high purity nitrogen gas, which was used as the carrier gas and purging gas at a flow rate of 20 sccm. Typical ALD deposition sequences were DEZn (20 ms)/N_2_ purge (20 s)/H_2_O (20 ms)/N_2_ purge (20 s) and (TEMAZ (150 ms)/N_2_ purge (25 s)/H_2_O (20 ms)/N_2_ purge (25 s) for the growth of ZnO and ZrO_2_ films, respectively. The sandwich structural ZnO:Zr films were obtained by repeating N cycles of DEZn/H_2_O followed by one cycle of TEMAZ/H_2_O, then M cycles of DEZn/H_2_O to get access to the desired doping concentration, as shown in Figure 8. The temperature for depositing the ZnO:Zr films was maintained at 200 °C. The thicknesses of the films were measured by ellipsometer and were in the range of 94–95 nm. The prepared ZnO:Zr films were subjected to RTA (RTP-600XP, Modular Process Technology Corp., San Jose, CA, USA) treatment in the temperature range of 350–950 °C (with 200 °C as a step) in a high purity nitrogen atmosphere for 3 min. Meanwhile, RTA treatment at 850 °C also carried out as the resistivity increased rapidly during 750–950 °C.

The structural and crystallinity characterizations of the prepared samples were performed by an X-ray diffraction (XRD) system (D8 Advance, Bruker, Billerica, MA, USA) using Cu Kα (40 kV and 40 mA, λ = 1.54056 Å) on the range from 20° to 80° at 2 theta scale. The morphology were further characterized by AFM (Multimode 8, Bruker, Billerica, MA, USA). The optical properties of the annealing films were investigated using a UV–vis spectrometer (Cary 300, Agilent, Santa Clara, CA, USA). Electrical properties such as resistivity, carrier concentration, and mobility were measured by a Hall automatic measuring system (Accent HL6600PC, Midview City, Singapore) using the van der Pauw method. The contacts of the Hall measurement system were indium.

## 4. Conclusions

In conclusion, the structural, optical, and electrical properties of the ALD-grown ZnO:Zr films treated with various annealing temperatures have been examined. The XRD results show that the grain size increases at low annealing temperatures and decreases due to the crystal disorder caused by zirconium dioxide segregation. The AFM indicates that the RMS increases slightly when the annealing temperature is lower than 750 °C. Then, it increases rapidly at higher annealing temperatures. The UV–vis spectrum shows that the films have good average transmittance of over 92% in visible wavelength and the optical band gap of the films show red-shift with increases of the annealing temperature. The Hall measurement shows that the resistivity increases slightly before 550 °C due to the decrease of carrier concentration and the increase of the mobility. Then, all three parameters decrease rapidly at higher annealing temperature. It is demonstrated that ZnO:Zr films show good properties when treated in the temperature range between 350 and 550 °C.
